# Research Progress on Lignin Depolymerization Strategies: A Review

**DOI:** 10.3390/polym16172388

**Published:** 2024-08-23

**Authors:** Zhengfei Pei, Xiaofang Liu, Jiasheng Chen, Huan Wang, Hu Li

**Affiliations:** 1 Key Laboratory of Surveillance and Management, Invasive Alien Species in Guizhou Education Department, College of Biology and Environmental Engineering, Guiyang University, Guiyang 550005, China; pzfei86@163.com; 2 State Key Laboratory of Green Pesticide, Key Laboratory of Green Pesticide & Agricultural Bioengineering, Ministry of Education, State-Local Joint Laboratory for Comprehensive Utilization of Biomass, Center for R&D of Fine Chemicals, Guizhou University, Guiyang 550025, China; gs.jschen22@gzu.edu.cn (J.C.); gs.hwang23@gzu.edu.cn (H.W.)

**Keywords:** aromatic biopolymers, lignin depolymerization, photocatalysis, electrocatalysis, enzymes

## Abstract

As the only natural source of aromatic biopolymers, lignin can be converted into value-added chemicals and biofuels, showing great potential in realizing the development of green chemistry. At present, lignin is predominantly used for combustion to generate energy, and the real value of lignin is difficult to maximize. Accordingly, the depolymerization of lignin is of great significance for its high-value utilization. This review discusses the latest progress in the field of lignin depolymerization, including catalytic conversion systems using various thermochemical, chemocatalytic, photocatalytic, electrocatalytic, and biological depolymerization methods, as well as the involved reaction mechanisms and obtained products of various protocols, focusing on green and efficient lignin depolymerization strategies. In addition, the challenges faced by lignin depolymerization are also expounded, putting forward possible directions of developing lignin depolymerization strategies in the future.

## 1. Introduction

Over the past century, global economic growth, urbanization, and population growth have largely relied on the exploitation and utilization of limited fossil fuels. For example, global energy consumption increased by 2.3% in 2018, almost twice the average growth rate since 2010. Due to the continued growth in fossil fuel consumption, global carbon dioxide emissions have climbed to 33.1 GT, exacerbating serious environmental problems such as global warming and climate change [1]. With the gradual depletion of fossil fuels, the world is paying increasing attention to environmental protection and resource conservation, and renewable energy has become an urgent need to achieve sustainable development [2]. Lignocellulosic biomass (LB) is regarded as an ideal substitute for fossil-based carbon resources due to advantages such as environmental protection, sustainability, and low cost, and it is widely used in the production of biofuels and valuable chemicals [3]. Lignocellulosic biomass is mainly composed of three polymeric components, cellulose, hemicellulose, and lignin, of which hemicellulose (accounting for 20–30%) and cellulose (accounting for 40–50%) are polymers of C_5_ and C_6_ sugars, respectively [4]. Currently, most advanced biofuel production strategies focus on the conversion of cellulose and hemicellulose, which have been intensively studied and have given rise to mature technologies in the industrial production of biofuels and important chemicals such as bioethanol, 5-hydroxymethylfurfural, furfural, and levulinic acid [5]. However, the utilization rate of lignin is relatively low, which has attracted increasing attention.

Lignin is the second most abundant natural biopolymer on the Earth, only inferior to cellulose [6]. It is also the largest renewable source of aromatic biopolymers, constituting 15–40% of the dry weight of most plants. Its structure is three-dimensional and of a highly cross-linked macromolecular nature, composed primarily of phenylpropane (C_6_–C_3_) units with phenolic hydroxyl and methoxy substituents. In its backbone, there are three different types of phenylpropane monomer units, including p-hydroxyphenyl (H), guaiacyl (G), and syringyl (S) fragments. Lignin contains a large number of functional groups and chemical bonds, and the complex combination of these bonds and groups makes its structure highly recalcitrant and heterogeneous [7]. As a result, lignin is often not effectively utilized in most biorefining processes due to its recalcitrant and heterogeneous properties. Currently, the global papermaking industry processes a large amount of lignocellulosic biomass each year, generating approximately 150–180 million tons of lignin as an industrial byproduct, but the utilization rate is less than 2%. Most lignin is considered waste and is discarded or used as low-value fuels, which not only wastes resources but also causes serious environmental pollution. Therefore, for the sustainable development of a biorefining strategy, it is particularly important to efficiently convert renewable lignin into high value-added chemicals and biofuels. This conversion not only contributes to the deep recycling of resources, but is also an important measure towards achieving a green economy and reducing dependence on fossil fuels.

With the deepening of research in related fields and the continuous advancement of technology, the high-value utilization of lignin has made significant progress. Nowadays, three development strategies have been adopted for the high-value transformation and utilization of lignin: (i) lignin fractionation technology, (ii) lignin depolymerization and conversion, and (iii) lignin modification and upgrading [8]. The key to these strategies is to break the stubbornness and structural heterogeneity of lignin itself by regulating the breaking of chemical bonds and optimizing the modification of functional groups. In particular, in the process of lignin depolymerization and further conversion, the main goal is to reduce the molecular weight of macromolecular lignin, thereby generating liquid bio-oil rich in monomeric aromatic compounds (MACs) and low-molecular-weight products such as dimers, trimers, and polymers [9]. During the depolymerization process, the aromatic ether bond is prone to break [10], and the released depolymerization products have great potential as biofuels, bio-based chemicals, and advanced biomaterials. At present, pyrolysis is one of the main technologies for lignin depolymerization, and its effect is significantly affected by the operating temperature. The degree of lignin depolymerization varies at different temperatures. Temperature plays a vital role in promoting the breaking of C–C bonds [11]. In the field of chemical catalytic lignin depolymerization, the research focuses on the design of new catalysts and the development of green solvents [12]. In addition, with the improvement in people’s environmental awareness and the recognition of the high added value of lignin depolymerization products, biological depolymerization, photocatalysis, and electrocatalysis strategies have also been deeply studied and promoted, showing broad development prospects [13,14,15]. These achievements not only enrich the value chain of lignin products, but also lay a solid foundation for the comprehensive development and sustainable utilization of biomass resources, making important contributions to promoting the development of a green and circular economy.

In this *review*, the depolymerization strategies for lignin, which are currently receiving much attention, are comprehensively introduced. Different from previous research reports [15,16,17], this review pays special attention to the up-to-date green, environmentally friendly, and economical depolymerization methods that have emerged in recent years, such as deep eutectic solvent (DES) technology, photocatalysis, and electrocatalysis, for high-performing lignin depolymerization. In addition, the current status of the combined use of various strategies is also introduced, and insights into the future development and direction of lignin depolymerization strategies are proposed, providing support for the high-value application of lignin.

## 2. Lignocellulosic Structure

**Figure 1:**
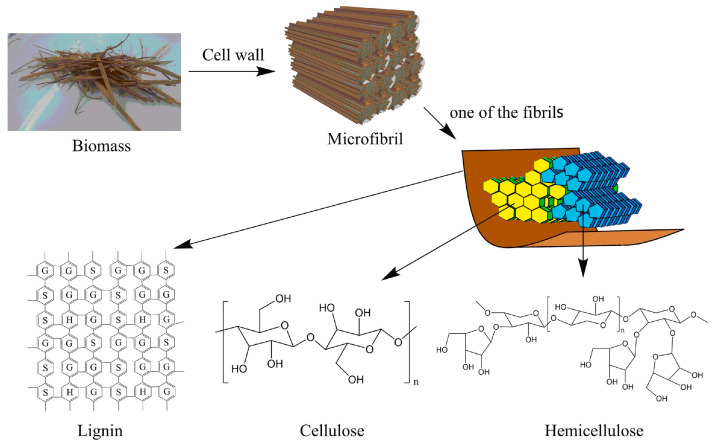
Lignocellulose in biomass and its composition [15].

Of the three main components of lignocellulosic biomass, cellulose and hemicellulose are two major carbohydrates that are tightly bound to lignin, forming a complex and stubborn structure (Figure 1) [15]. Cellulose is the main component of lignocellulose and is composed of glucose molecules linked by β-(1–4)-glycosidic bonds [18]. The polysaccharides in the biomass are linked to each other by hydrogen bonds, which help cellulose to crystallize and resist degradation, thus making subsequent separation processes difficult. Cellulose can be hydrolyzed into glucose by enzymatic hydrolysis, which can be further converted into high-value compounds. Hemicellulose is the second most abundant component in lignocellulose. Hemicellulose is linked to cellulose by hydrogen bonds and van der Waals interactions, which enable hemicellulose to form a highly resistant network with cellulose [19]. Unlike cellulose, hemicellulose consists of short, highly branched polymers of five- and six-carbon polysaccharide units, such as xylan, mannan, β-glucan, and xyloglucan. The highly branched and amorphous nature of hemicellulose makes it easy to convert. Compared with cellulose and lignin, hemicellulose has a much lower degree of polymerization, making it less stable than cellulose [20].

### Lignin Structure, Properties, and Classification

**Figure 2:**
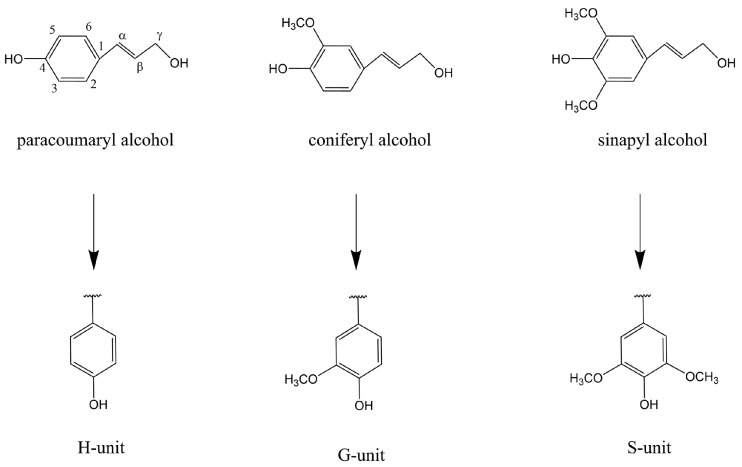
The precursors and basic unit in lignin molecule [15].

Lignin is the most complex natural polymer, which contains a mixture of aromatic and aliphatic components (Figure 2) [15]. Its structure is a three-dimensional network in which different phenylpropane units are randomly connected to each other through ether bonds (C–O) or carbon–carbon bonds (C–C). Lignin molecules are rich in various functional groups, such as phenolic hydroxyl groups, alcoholic hydroxyl groups, carbonyl groups, methoxy groups, carboxyl groups, and conjugated double bonds. These functional groups give lignin excellent chemical reactivity, enabling it to participate in a variety of chemical reactions such as oxidation, reduction, hydrolysis, and sulfonation. With the modification of these functional groups, the scope of applications of lignin can be further broadened.

**Figure 3:**
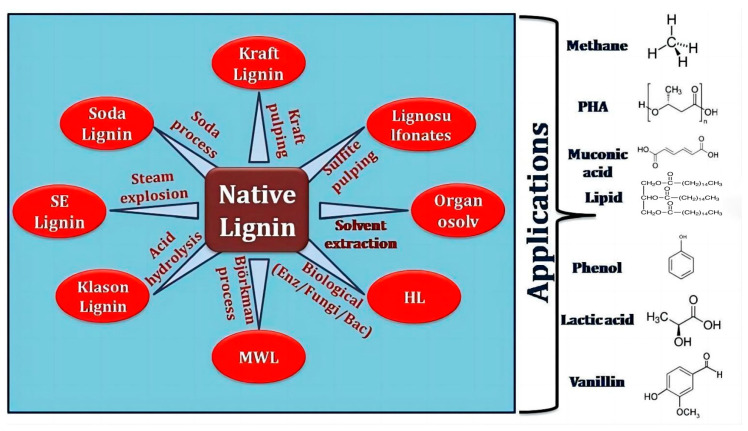
Different lignin extraction or separation processes and common applications of lignin [14].

Lignin can be classified into two categories, natural lignin and industrial lignin, based on the classification of sources and treatment methods [21]. Natural lignin refers to unmodified lignin that retains the original structure of lignocellulose. It does not exist independently in nature, but is a component of lignocellulose complexes [22]. After a series of chemical and mechanical treatments, lignin is separated as a by-product and has a structure that varies with the extraction process, and it is generally called industrial lignin [23]. There may be significant differences between industrial lignin and natural lignin in terms of the abundance of C–O and C–C bonds, which significantly affects the subsequent depolymerization options [24]. The naming of industrial lignin is usually based on its separation or extraction method (Figure 3), such as kraft lignin, which comes from the kraft paper production process of the paper and pulp industry, while lignin sulfonates are mass-produced by the sulfite pulping process. Kraft lignin needs to be modified to enhance its reactivity due to its hydrophobicity and low reactivity, while lignin sulfonates are soluble in water and have a high molecular weight due to the hydrophilicity of the sulfonate group and their low phenol content [25]. In addition, lignin extracted from biomass by specific organic solvents (such as acetic acid, ketones, esters, etc.) is called organosolv lignin [26]. Compared with other lignins, organosolv lignin has a higher purity and shows great potential in biofuel production, but its application is limited due to the high production costs [27]. An in-depth exploration of the structural characteristics of lignin and its potential for depolymerization into monomers is crucial to building a sustainable biorefining system [20].

## 3. Thermochemical Depolymerization of Lignin

In the depolymerization process of lignin, the breaking of chemical bonds is crucial, and temperature is one of the key factors that affects the breaking of these bonds [28]. Therefore, increasing the temperature has become an important means to promote the depolymerization of lignin. Common strategies for the thermochemical depolymerization of lignin include pyrolysis, hydrogenolysis, and hydrolysis [29]. The notable feature of these methods is that lignin can be effectively depolymerized regardless of the presence of a catalyst. In addition, the depolymerization efficiency of lignin can be significantly improved by combining thermochemical depolymerization technology with physical auxiliary means, such as combining ultrasound technology with pyrolysis [30]. However, the thermochemical depolymerization of lignin still faces many challenges, such as harsh reaction conditions, a high coke yield, low depolymerization selectivity, and difficulty in separating the final compounds [31].

### 3.1. Pyrolysis

**Figure 4:**
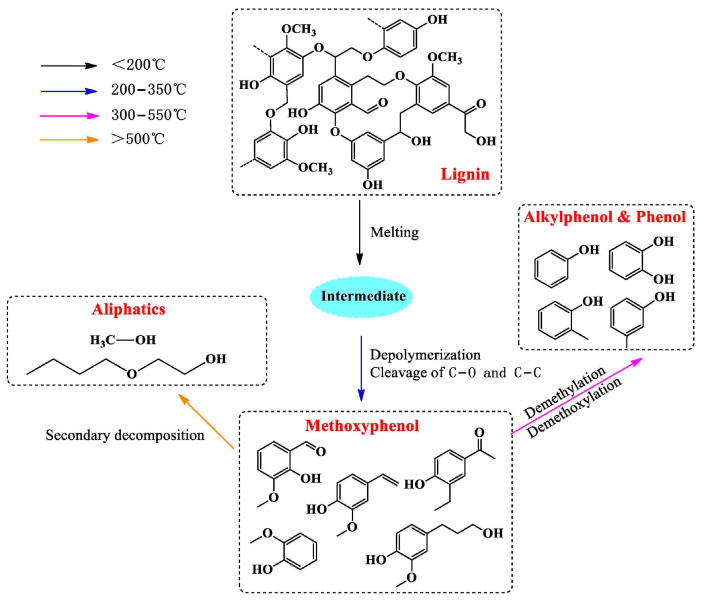
Principle of lignin pyrolysis [32].

As one of the effective thermochemical strategies for the depolymerization of lignin, pyrolysis technology has attracted much attention [32]. This technology mainly involves the heat treatment of lignin under anaerobic or low-oxygen conditions at high temperatures of ca. 150–800 °C [33]. The principle of lignin pyrolysis is shown in Figure 4. In this process, catalysts can be selectively used to promote the decomposition of lignin into a variety of products, including carbon monoxide (CO), carbon dioxide (CO_2_), water vapor (H_2_O), gaseous hydrocarbons, volatile liquids, monomeric lignin, monomeric phenols, and various polysubstituted phenols. At the same time [34], thermally stable char, coke, and other by-products are also produced. It is worth noting that the introduction of oxygen can accelerate the depolymerization rate of lignin, while in an anaerobic environment, the breaking of chemical bonds is relatively slow, and the depolymerization process has difficulty in producing further carbon dioxide. The products obtained after lignin depolymerization are rich in various aromatic monomers, showing that pyrolysis technology has great potential as an effective way to convert lignin and biomass resources into new biomaterials [35]. Generally, the specific composition of the depolymerization product and the yield of each component are profoundly affected by the source of lignin and its separation and purification method [36]. To optimize the depolymerization effect in the pyrolysis strategy, the molecular weight of the depolymerized lignin fragments is often reduced by increasing the severity of the reaction conditions (e.g., increasing the temperature and extending the residence time of the reactants) [37]. In addition, adjusting external factors, such as the type of catalyst and reaction time, is also an effective way to improve depolymerization efficiency and regulate product distribution.

As the temperature rises, different chemical bonds in the lignin structure can be broken one after another, so multiple reactions will occur during the entire lignin thermal depolymerization process, which makes it difficult to clearly classify the pyrolysis stage. To simplify the understanding, most pyrolysis processes are divided into two stages according to differences in the lignin depolymerization products [38]. The main pyrolysis process of lignin occurs in these two stages. When the pyrolysis reaction temperature is in the range of 150–400 °C, it belongs to the first stage of pyrolysis. In this process, due to the relatively high stability of aromatic methylation groups and condensation bonds, the depolymerized lignin fragments produced in this stage are mostly basic units of lignin, such as 4-methylguaiacol, eugenol, and coniferyl alcohol, as well as some chemicals derived from lignin, such as vanillin, isoeugenol, and certain unsaturated alkyl groups. It is particularly worth mentioning that vanillin is converted from lignin and has shown great potential for commercialization. In the second stage of pyrolysis, most of the ether bonds will be broken, including β-O-4 bonds, which are the most abundant bonds in lignin and account for more than 60% of the total bonds in hardwood lignin. The breaking of β-O-4 bonds is crucial to improving the quality of lignin depolymerization products.

When lignin is pyrolyzed, the formation of coke may reduce the yield of depolymerized monomers. The coke formation process can also be divided into two main stages. The first stage is at 450 °C when most of the coke is converted from eugenol. When the temperature exceeds 550 °C, it enters the second stage of coke formation, and the methoxy group in guaiacol becomes the main cause of coke formation. In addition, some studies have pointed out that the increase in the number of methoxy groups is an important factor leading to coke formation, which is also a key problem that needs to be solved by current pyrolysis technology.

### 3.2. Microwave-Assisted Lignin Depolymerization

Microwave technology has gradually become a viable alternative to traditional heating and has seen great achievements in the field of value-added biomass conversion. Compared with traditional technologies, microwave pyrolysis is an emerging technology that uses microwaves as a heat source to thermally convert biomass and relevant wastes. The microwave-assisted pyrolysis of biomass can not only reduce the required pyrolysis temperature, but also change the chemical properties of the product. Zhu et al. [33] showed that the pre-methylation of benzyl alcohol using microwave technology can effectively promote the depolymerization of lignin into aromatic monomers and reduce the oxygen content of these monomers. Liu et al. [34] explored the process of lignin degradation into aromatic compounds in isopropanol under microwave heating, revealing that lignin first decomposes into oligomers and then hydrogenates into monomers. At the same time, it was found that increasing the reaction temperature will also promote the polymerization of monomers into oligomers again. Dhar et al. [39] showed that the use of microwaves and organic solvents for lignin value-added treatment can be an effective means to produce high-value chemicals under mild conditions. Unlike traditional external heating methods [40], microwave-assisted pyrolysis relies on the interaction between microwaves and the internal dipoles of the target material to generate heat, which can achieve uniform and efficient heating inside the material, avoiding overheating and a prolonged reaction time. This feature is particularly important for processing biomass feedstocks with poor thermal conductivity [41]. Microwave technology can also work synergistically with the polymerization process of lignin to increase the yield and selectivity of the product. Duan et al. [42] significantly improved the yield and quality of bio-oil by the co-pyrolysis of lignin and polypropylene with microwave-assisted catalysis. The main components, such as aromatics and cycloalkanes, can also be used as potential raw materials for aviation fuel. In most cases, the microwave-assisted pyrolysis of lignin is not used alone, but as a pretreatment technology before depolymerization, combined with a catalyst to achieve a more selective reaction. For example, using iron sulfate as a catalyst, under the assistance of microwaves, the C_α_-C_β_ bonds in phenolic and non-phenolic lignin model dimers can be selectively broken, thereby effectively depolymerizing the lignin sample into aromatic monomers [43]. In addition to promoting the depolymerization of lignin, microwave technology can also assist the liquefaction or dissolution of lignin by accelerating the heating rate. Muley et al. [44] showed that low eutectic solvents can effectively and selectively dissolve lignin during biomass fractionation. Microwave heating enhances this process through faster heating rates. Analysis shows that the molecular weight distribution of microwave-treated lignin is relatively narrow compared to traditional heating [45]. Certain lignin bonds are stretched under microwave radiation, which increases the probability of their breaking [46]. Based on this study, microwave heating can be effectively used for biomass deconstruction and lignin depolymerization in low eutectic solvents, thereby reducing the lignin depolymerization time and enhancing the selectivity of the obtained products.

Overall, among pyrolysis strategies, single high-temperature pyrolysis does not have an advantage in industrialization due to its high energy consumption and complex by-products. Microwave, as an assisted depolymerization technology, has the advantages of clean, efficient, and controllable features. A tandem depolymerization strategy with microwave-assisted pyrolysis shows great potential in industrialization.

## 4. Chemocatalytic Depolymerization of Lignin

In recent years, the research focus of chemocatalytic lignin depolymerization has been mainly on the development of new catalysts and the design of environmentally friendly solvents. According to previous reports, chemical catalysts used for lignin depolymerization can be roughly divided into four types: (i) acid catalysts, (ii) base catalysts, (iii) metal catalysts, and (iv) ionic liquid-based catalysts [47]. Compared with thermal depolymerization strategies, chemocatalytic lignin depolymerization strategies show significant advantages in terms of a lower energy consumption, milder reaction conditions, and better product separation performance. However, the use of chemical catalysts also has the potential risk of environmental pollution. In addition, low eutectic solvents have become a hot topic of current research due to their excellent lignin solubility and environmental protection characteristics. To improve the efficiency of lignin depolymerization and product purity, these catalysts can be combined to play a role in different depolymerization stages.

### 4.1. Acid or Base Catalysts

Acid-catalyzed lignin depolymerization is a long-standing and classic method [48], and its application can be traced back to 1943. It is one of the important technologies in this field. In the process of lignin depolymerization, strong Brønsted acids such as sulfuric acid (H_2_SO_4_) and hydrochloric acid (HCl), as well as relatively weak phosphoric acid (H_3_PO_4_), play a key role in effectively promoting the cleavage of phenyl glycosidic bonds and β-O-4 ether bonds [49]. Past studies have shown that the introduction of dilute sulfuric acid as a catalyst in a 1:1 mixed solvent system of ethanol and water can achieve a lignin depolymerization efficiency of up to 70%, while maintaining the rich content of hydroxyl and carbon in the product [50]. However, although strong acid catalysts show significant depolymerization effects, their harsh reaction conditions and high costs limit the further development of acid-catalyzed depolymerization technology. Recently, *p*-toluenesulfonic acid (p-TsOH) has attracted attention due to its excellent lignin solubility and has shown a good delignification performance in lignocellulose pretreatment. Although its direct depolymerization effect needs to be improved, it provides a potential research direction for the development of low-cost acid-catalyzed depolymerization technology. It should be noted that although acid-catalyzed depolymerization technology has been widely studied, it usually requires a high temperature, high pressure, and long reaction time, which may not only damage the reaction equipment but also cause environmental pollution problems. In addition, the repolymerization that may occur during the depolymerization process will also reduce the yield and purity of the target product.

Similar to the acid-catalyzed mechanism, base catalysis has been deeply studied in the field of lignin depolymerization, with wide discussion of its depolymerization effect. Base catalysts can effectively promote the breaking of ether bonds in lignin, thereby achieving a more thorough depolymerization. In this field, NaOH and KOH are widely used as typical strong base catalysts. In particular, NaOH is favored in industrial applications because of its high cost-effectiveness and good depolymerization effect, which can significantly increase the yield of bio-oil. The addition of NaOH can increase the amount of lignin depolymerization products [51]. The use of strong bases (e.g., KOH and NaOH) can not only promote the conversion of lignin, but also increase the diversity and quantity of depolymerization products, while weak bases such as Ca(OH)_2_ do not have this effect. In addition, all the base catalysts, used effectively, can reduce the formation of coke and promote the decomposition of lignin into monomer compounds such as phenol, o-cresol, p-cresol, m-cresol, guaiacol, and catechol, while maintaining the reactivity of phenolic substances during the depolymerization process [52]. However, when the concentration of the alkali catalyst is too low, the depolymerization effect of lignin is not ideal. Therefore, many studies began to explore the use of organic solvents as an alternative, but this also raised new problems. For instance, organic solvents may undergo condensation reactions with lignin molecules [53]. In addition, the carboxylic acids released during the depolymerization process will lower the pH value of the reaction system, thereby exacerbating the re-condensation phenomenon [54]. Therefore, the repolymerization phenomenon observed during the base-catalyzed depolymerization process poses a challenge to the high-value utilization of lignin. Whether using strong or weak base catalysts, it is difficult to obtain a single or specific product after the reaction. Similar to acid catalysis, the use of base catalysts also faces common problems such as strict reaction conditions and high costs.

### 4.2. Metal Catalysts

Given that acid/base catalysts have poor selectivity in the process of lignin depolymerization, it is difficult to generate specific products in a targeted manner, and strict reaction conditions are required. Therefore, it is particularly important to explore a highly selective and mild lignin depolymerization strategy, and metal catalysts have become the focus of research. Metal catalysts are a common means for the catalytic depolymerization of lignin, including transition metal catalysts, precious metal catalysts, and bimetallic catalysts. Transition metals such as Fe, Ni, Cu, Co, and Mo are widely used in catalytic lignin depolymerization because of their low price, high efficiency, and abundant reserves. Taking nickel as an example, it has great potential in the production of phenolic chemicals. It can specifically cleave ether bonds and specifically hydrolyze the carbon hydroxyl groups on the side chains of lignin into alkanes [55]. In addition to transition metals, precious metal catalysts have also attracted much attention, mainly including platinum group metals such as Ru, Rh, Pd, and Pt.

The selection of metal carriers is crucial to the catalytic depolymerization of lignin. The carrier is designed to optimize metal dispersion, improve catalytic efficiency, prevent metal agglomeration, and contribute to catalyst stability and metal recovery. Activated carbon, silica, alumina, zeolite, etc., are commonly used carriers. For example, activated carbon-supported Ru can significantly improve the bio-oil yield with the assistance of formic acid, showing excellent lignin conversion activity [56]. In addition, through comparative experiments, it was found that during the hydrogenation treatment of the lignin model compound eugenol, the activity was highly dependent on the type of metals. For example, Ru/C had the lowest ratio of hydrogenation/deoxygenation products, while Pd/C had the highest [57]. Although precious metals can increase the bio-oil yield and reduce the O/C ratio, thereby improving the depolymerization of lignin, their scarcity and high cost are significant disadvantages [58].

In order to reduce costs, bimetallic catalysts composed of transition metals and precious metals or transition metals have been widely used in the field of lignin depolymerization and conversion. For example, nickel is favored for its ability to efficiently decompose C–O bonds, and it can be combined with other metals to form bimetallic alloys. It is reported that under mild conditions, the use of Ni-Au bimetallic catalysts for lignin depolymerization can achieve depolymerization monomer in a yield of 14 wt%, which is three times that of pure Ni catalysts [59]. Although bimetallic catalysts can improve the reaction selectivity and effects, they also have limitations. For example, the presence of precious metals will increase costs, and may lead to the excessive hydrogenation of aromatic compounds and reduce the yield of phenolic products.

In summary, the future development of metal catalysts will pay more attention to cost control, including the use of low-cost metals to replace precious metals or the development of bimetallic catalysts, and strive to improve the reuse efficiency of the catalysts.

### 4.3. Ionic Liquids

Ionic liquids are composed of large asymmetric organic cations and smaller organic or inorganic anions. They have a melting point below 100 °C and can remain in the liquid state at room temperature [60]. This special salt material has excellent chemical and thermal stability, and excellent electrical conductivity, which can effectively improve the solubility of various solvents [61]. It is worth mentioning that a significant advantage of ionic liquids is their designability. Their physical and chemical properties can be customized by adjusting the cations and anions in the salt [62].

The anions in ionic liquid can stabilize the intermediates in the depolymerization process of lignin, thus significantly affecting the yield of the final monomer product [63]. Tolesa et al. [64] used two ammonium-based ionic liquids, diisopropylethylammonium acetate (DIPEAA) and diisopropylethylammonium octanoate (DIPEAO), to study the depolymerization of alkaline lignin. These experiments were carried out at different temperatures and reaction times, and the results confirmed that in the aqueous solution environment of these two ionic liquids, alkaline lignin can be successfully depolymerized to produce phenolic products. During this process, the anions of the ionic liquid coordinate with lignin to promote the breaking of chemical bonds. At the same time, the hydrophobic nature of the alkyl part of the ionic liquid may also promote the depolymerization reaction. In the depolymerization process of lignin, ionic liquids play a dual role, acting as both solvents and catalysts, accelerating the depolymerization of lignin. In addition, ionic liquids can also be used in combination with metal catalysts (e.g., cobalt, manganese, and copper) to jointly promote the depolymerization of lignin. The combination of manganese nitrate and 1-ethyl-3-methylimidazolium trifluoromethanesulfonate can convert more than 63% of lignin into 2,6-dimethoxy-1,4-benzoquinone under certain conditions, with a yield of 11.5% [65].

It is worth noting that ionic liquids also have some limitations. They tend to interact with lignin-derived aromatic compounds, making it difficult to separate ionic liquids from monomer products. At the same time, their high cost, low recovery rate, and limited reusability also limit the widespread application of ionic liquids [66]. In addition, the mechanism of ionic liquid’s degradation of lignin is currently unclear, which makes the qualitative and quantitative analysis of lignin degradation products complicated and becomes an obstacle to the further development of ionic liquids. Therefore, the research and development of green, efficient, and low-cost ionic liquid catalyst systems, or the exploration of other more environmentally friendly alternative solvent systems, are of great significance for the conversion and value-added utilization of lignin.

### 4.4. Deep Eutectic Solvents

Deep eutectic solvent (DES), a liquid solvent formed by the eutectic mixture of hydrogen bond acceptors (HBA) and hydrogen bond donors (HBD), is known as a true green solvent for its low vapor pressure, excellent thermal stability, low toxicity, and biodegradable and environmentally friendly properties [4]. At the same time, it has also become the preferred solvent in the field of lignocellulosic biomass processing. DES can not only effectively dissolve lignin in lignocellulose, but it can also achieve the efficient extraction of lignin while maintaining its original structure to the greatest extent. Under certain conditions, DES can even give lignin better upgraded properties.

At present, DES is widely used in the pretreatment of lignocellulose. In this process, biomass mainly undergoes the dissociation of aromatic ether bonds. It is worth mentioning that the lignin separated from DES has a small molecular weight and is evenly distributed, which is very similar to the lignin in the original plant. It has been pointed out that the depolymerization of lignin can be achieved by using a new ternary DES [67]. For example, by adding ethylene glycol (EG), the re-condensation of lignin during the depolymerization process can be effectively prevented, thereby ensuring that the obtained lignin retains a high β-O-4 content [68]. This feature provides great convenience for the subsequent depolymerization of lignin into various phenolic products. In addition, the combination of DES and microwave technology has performed well in the fractionation treatment of biomass and the depolymerization of lignin. Compared with traditional DES heating technology, the microwave-assisted DES depolymerization of biomass can more selectively promote the breaking of chemical bonds, thereby significantly shortening the reaction time, which is of great significance for industrial applications [44]. On the other hand, since DES can dissolve lignin and have a good electrochemical conductivity, it is often used as an ideal reaction medium in the electrochemical depolymerization of lignin [69]. According to the relevant reports, Di Marino et al. [70] used DES as a reaction medium for electrochemical reactions, achieved the depolymerization of kraft lignin, and extracted the products by liquid–liquid extraction. The two most abundant products obtained after depolymerization are guaiacol and vanillin.

Given the wide application of DES in biomass pretreatment and its excellent solubility and extraction ability for lignin, using DES can be considered as an electrochemical reaction medium. In the future, comprehensive reaction systems can be designed that will cover biomass pretreatment and lignin depolymerization, and integrate waste utilization and DES recovery, thereby achieving a seamless connection between upstream biomass processing and downstream technologies. However, understanding the reaction mechanism of the DES-based electrochemical depolymerization of lignin is not enough, and at the same time, DES-compatible and active catalysts/electrodes have not been fully developed. Therefore, more research efforts in these areas are needed to promote the development of DES-based electrochemical lignin depolymerization technology.

Among chemical catalytic depolymerization strategies, the traditional acid/base depolymerization strategy has high efficiency, but the equipment requirements are relatively stringent. Metal catalysts cannot be widely used due to their high cost. Ionic liquids show high solubility in lignocellulosic biomass, but are also difficult to develop due to cost factors. As a green solvent, low eutectic solvents have attracted widespread attention due to their strong designability and low-cost characteristics, but the research in related fields is not yet in-depth. A single chemical depolymerization strategy cannot meet the needs of industrial production. The development of an efficient and low-cost depolymerization system is a challenge that needs to be faced in the future.

## 5. Photocatalytic Lignin Depolymerization

In recent years, using solar energy with a variety of photocatalysts to enhance the utilization value of biomass has attracted increasing attention [71]. The so-called photocatalytic depolymerization/conversion strategy of lignin refers to the process of directly or indirectly depolymerizing/converting lignin through photogenerated carriers generated by catalysts under the excitation of a specific light source [72]. Compared with the traditional pyrolysis method, photocatalytic technology uses light energy as the driving force, allowing the reaction to proceed under milder conditions. In addition, photocatalysis also has the advantages of low cost and low environmental pollution, thus achieving a good balance between economic benefits and environmental protection [73]. In addition, due to the non-equilibrium distribution of energy in the photocatalytic system, this technology can selectively break the C–O/C–C bonds in lignin, thereby producing high-value-added chemicals or advanced biofuels. Therefore, photocatalysis has great potential in depolymerizing lignin into valuable products [74].

### 5.1. Sole Photocatalysis

In the process of photocatalytic lignin depolymerization, the role of photocatalysts is crucial. At present, the photocatalysts used in this process can be roughly divided into two types: heterogeneous and homogeneous catalysts. Heterogeneous photocatalysts typically cover metal oxides, metal sulfides, carbon-based materials, and heterojunction materials. These materials occupy an important position in the field of photocatalytic lignin depolymerization due to their low cost, excellent stability, and easy separation from the depolymerization products. Homogeneous photocatalysts are mainly composed of organometallic complexes formed by the combination of metal ions and organic ligands. This type of catalyst allows us to enhance its catalytic activity by fine-tuning the metal ions and organic ligands. However, in the actual application of lignin depolymerization, the use of homogeneous catalysts is not widespread, mainly due to the difficulty in separating the catalytic system from the final product. Despite this, homogeneous catalysts show extremely high selectivity during the reaction. Looking forward to the future, if a way to solve the problem of separating the products from the catalytic systems can be found, the application of homogeneous catalysts will usher in a broader development space.

In the family of heterogeneous catalysts, metal oxides are highly respected for their excellent photostability, low price, and strong oxidation ability, which have been widely used in the field of photocatalysis [75]. In particular, TiO_2_, as the most intensively studied photocatalyst, has shown excellent application potential in the photocatalytic decomposition of H_2_O to produce hydrogen and the degradation of pollutants to protect the environment. However, the traditional metal oxide catalyst TiO_2_ is difficult to effectively excite by sunlight due to its high energy band gap value, and can only absorb and utilize less than 4% of the solar energy spectrum [76]. This limitation greatly restricts the application scope of TiO_2_ in selective photocatalysis. To break through this bottleneck, researchers are committed to modifying TiO_2_ to improve its catalytic activity. For example, Xiao et al. [77] successfully developed a TiO_2_ microsphere with an oxygen defect structure on the surface. They used 200 nanometers of amorphous SiO_2_ to regionally modify it and tried to apply it to the depolymerization process of lignin. This SiO_2_ surface modification not only significantly enhances the adsorption capacity of lignin, but also achieves the efficient utilization of light per unit area by effectively refracting the incident light, thereby greatly improving the depolymerization efficiency of lignin. In addition to TiO_2_, ZnO is also a commonly used metal oxide catalyst in the field of photocatalysis. Xu et al. [78] successfully prepared Ni-doped ZnO containing oxygen vacancies by hydrothermal reaction. The doping of Ni ions reduced the band gap of ZnO and enhanced its absorption capacity for visible light. At the same time, the doping of Ni ions also increased the concentration of oxygen vacancies, which improved the separation efficiency of the photogenerated electrons and holes to a certain extent. Sodium lignin sulfonate was successfully depolymerized into vanillin, vanillic acid, other organic matter, CO_2_, and H_2_O, among which the yields of vanillic acid and guaiacol were 9.3% and 1.5%, respectively, under optimal conditions. The doping of Ni ions promoted the formation of •O_2_^−^, and more oxygen vacancies could also increase the concentration of holes and •OH, thereby decomposing sodium lignin sulfonate into CO_2_ and H_2_O.

**Figure 5:**
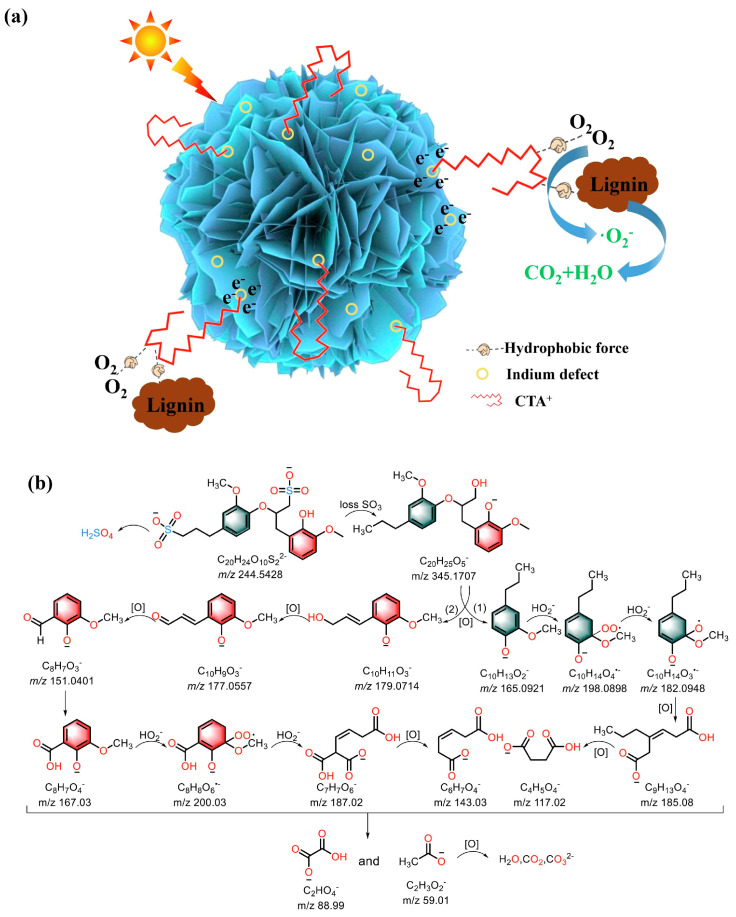
Depolymerization principle of In_x_S_3_-C catalyst. (**a**) Schematic illustration of lignin degradation over InxS3-C. (**b**) Degradation mechanism and degradation pathways of lignosulfonate [80].

Compared with metal oxide catalysts, metal sulfide catalysts have a smaller band gap width, allowing them to be excited by visible light in sunlight. As a result, metal sulfides show excellent catalytic activity in visible light-driven catalytic reactions. Indium sulfide (In_2_S_3_) is a typical narrow-band-gap semiconductor material. Its unique light absorption properties extend to the visible and near-infrared regions, which makes it have a great application potential in the field of biomass conversion. Experiments have shown that In_2_S_3_ nanoparticles can effectively photodegrade β-O-4 lignin model compounds to generate aromatic monomers, including value-added acetovanillin [79], vanillin, and coniferyl aldehyde. However, the original In_2_S_3_, like other original metal sulfides, also faces problems such as poor stability, a susceptibility to photocorrosion, and a high electron–hole recombination rate, which seriously affect its catalytic performance. Fortunately, the photocatalytic performance of metal sulfides can be effectively improved by adopting a modification strategy similar to that of metal oxides. Chen et al. [80] prepared a series of defective In_x_S_3_-C samples by adjusting the ratio of indium chloride tetrahydrate to thioacetamide, and inserting hydrophobic CTA^+^ at the indium defect position. The hydrophobicity of In_x_S_3_-C can be controlled by changing the defect concentration and the length of the CTA^+^ carbon chain. After irradiation with visible light for 30 min, In_0.75_S_3_-C can degrade 90% of lignin sulfonate. Indium defects increase the absorption range of visible light and improve the separation ability of the photogenerated carriers. In addition, CTA^+^ increases the hydrophobicity of the catalyst, adsorbs lignin sulfonate and oxygen, and promotes mass transfer and surface reactions. Lignin is eventually decomposed into small molecules such as H_2_O and CO_2_. The relevant principle is shown in Figure 5.

In comparison with metal-based photocatalysts, carbon nanomaterials show a better light absorption ability, due to their unique properties of capturing and storing photogenerated electrons, which makes the photogenerated carriers have a longer survival time and is extremely beneficial for the photocatalytic conversion process of lignin. In particular, carbon nanomaterials (CNMs) have been widely used as catalysts or catalytic compositions in various driven reactions such as heat, electricity, and light. Among them, graphene has attracted much attention as a commonly used photocatalytic carbon nanomaterial. When graphene is hybridized with TiO_2_, the photocatalytic performance of TiO_2_ can be enhanced by significantly improving its charge transfer efficiency. In addition, graphite phase carbon nitride (g-C_3_N_4_) is also an excellent carbon-based photocatalyst [81]. It has good chemical stability and its preparation process is relatively simple. It is worth mentioning that g-C_3_N_4_ can specifically cleave the C_α_-C_β_ bond in the lignin structure. Compared with graphene, the electronic band structure of g-C_3_N_4_ is more conducive to the photocatalytic reaction, and its abundant electrons also help the migration of charges. Ku et al. [82] prepared mesoporous sulfur-doped carbon nitride (MSCN-0.5) by a simple one-step thermal condensation strategy. It was applied to the selective photocatalytic conversion of lignin to monophenolic compounds. It has a high activity and selectivity for the cleavage of C_α_-C_β_ bonds in β-O-4 lignin model compounds under visible light irradiation at room temperature. The visible light irradiation of β-O-4 lignin model compounds at room temperature can obtain aromatic aldehydes with a high yield of 98%. Mechanistic studies show that the C_β_-H bonds of lignin model compounds are activated by holes and generate key C_β_ radical intermediates, which further induce superoxide anion radicals (•O_2_^−^) to break C_α_-C_β_ bonds to generate aromatic oxygen-containing compounds. Waste oil tea shell (WCOS) was used as the research object to further understand its reaction mechanism on natural lignin. The final results showed that 33.2 mg of monophenolic compounds (including 22% vanillin and 34% syringaldehyde) could be obtained per gram of WCOS lignin.

In addition to carbon nanomaterials (CNMs), covalent organic frameworks (COFs), as an emerging carbon-based photocatalytic material, have also received widespread attention from the scientific research community in recent years [83]. The biggest advantage of COF materials lies in the orderliness of their structure and the ability to adjust the pore size according to actual needs. This flexibility enables COFs to make timely material adjustments based on the characteristics of selective catalytic reactions. However, the application of COFs in the field of lignin photocatalytic conversion is still in its infancy, and the main obstacles to their development are the instability of the materials and the relatively high cost.

**Figure 6:**
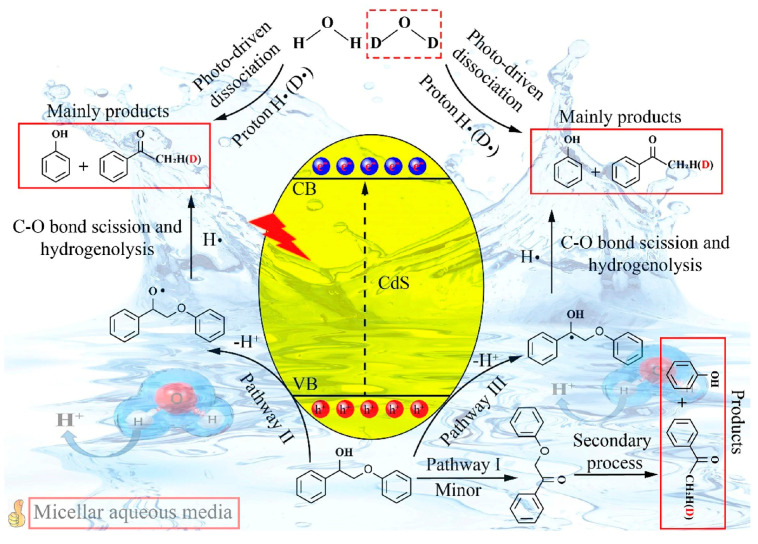
Mechanism diagram of photocatalytic cleavage of lignin C–O bonds [84].

Overall, different photocatalysts have their unique advantages and disadvantages. To overcome these inherent limitations and further enhance their advantages, various photocatalysts can be coupled to form heterojunction materials. This new type of heterojunction photocatalyst usually exhibits more significant advantages than a single photocatalyst, such as an extended carrier lifetime, improved solar energy absorption, more efficient separation and transport of electron–hole pairs, and controlled chemical potentials that are more suitable for the desired chemical reaction. At the same time, an environment conducive to the photocatalyst to exert its maximum catalytic performance can be created to achieve the maximum catalytic effect. For example, Xu et al. [84] used water as a hydrogen source to provide hydrogen in the process of the photocatalytic cleavage of C–O bonds, and successfully synthesized a micellar water medium composed of hexadecyltrimethylammonium bromide (CTAB) and water, which can effectively disperse lignin and provide hydrogen for the photocatalytic reaction (Figure 6). At the same time, the photocatalyst CdS was used to efficiently break the C–O bonds of lignin, and a high photocatalytic efficiency was obtained. Under optimal conditions, the photocatalyst CdS successfully depolymerized lignin to obtain ketones and phenolic compounds. Water can provide hydrogen for the photocatalytic cleavage of lignin C single bonds and O bonds, and a large amount of hydrogen plays a key role in improving photocatalytic efficiency. The proper inhibition of water on photogenerated holes can also effectively promote the cleavage of lignin C–O bonds. This study provides a new idea for the photocatalytic cleavage of lignin C–O bonds and promotes the high-value utilization of lignin. Dai et al. [85] synthesized a CdS/BiOI-V_I_ photocatalytic heterojunction containing iodine vacancies (V_I_) by the solvothermal method, and achieved an efficient conversion of sodium lignin sulfonate to vanillin under visible light irradiation. The experimental results show that the use of the CdS/BiOI-V_I_ heterojunction can obtain a higher vanillin yield. In addition, Cui et al. [86] also prepared a Z-type multi-component CdS/HPA-2/CN heterojunction photocatalyst by the hydrothermal method, and conducted in-depth research on its performance in the depolymerization of lignin into aromatic monomers. Their results showed that after 4 h of photocatalytic depolymerization, the total yield of vanillin and vanillic acid could reach 3.27%. More importantly, the authors also proved that after three consecutive cycles of use, the yield of aromatic monomers of this heterojunction catalyst could still remain at 81.0% of the initial value, showing good stability and reusability.

As mentioned above, the application of photocatalysts in the catalytic depolymerization of lignin has shown great development potential, but there are still some challenges to achieving industrial applications. First, the performance optimization of the catalyst is one of the core challenges. At present, photocatalysts face the problem of the rapid recombination of photogenerated electron–hole pairs, which seriously restricts the improvement of catalytic efficiency. In addition, the complex molecular structure of lignin requires photocatalysts to have a precisely regulated redox ability to effectively break specific chemical bonds. Therefore, the development and design of new photocatalysts with a high stability, strong redox ability, and an effective inhibition of electron–hole recombination is the key to improving catalytic performance. Secondly, precise control of the reaction conditions is crucial to optimize the catalytic effect. An uneven distribution of light intensity and slight changes in parameters such as the reaction temperature and pH value may have a significant effect on the depolymerization reaction of lignin. Therefore, the establishment of an advanced reaction condition control system to achieve the precise adjustment and stable maintenance of these parameters is of great significance for improving product selectivity, yield, and catalytic efficiency. In addition, the recovery and separation of reaction products is also a factor that needs to be considered. Finally, from an industrial perspective, photocatalytic lignin depolymerization technology still has problems such as a high catalyst cost, large equipment investment, and poor process stability.

### 5.2. Other Strategies of Assisted Photocatalytic Lignin Depolymerization

Although the photocatalytic depolymerization of lignin has achieved remarkable results, due to the extremely complex structure of lignin and its strong resistance, more methods are still needed for exploration to improve its depolymerization efficiency. Therefore, the organic integration of photocatalytic depolymerization strategies with other advanced technologies has become a direction worthy of in-depth exploration. Researchers such as Dhar et al. [79] innovatively combined commercial TiO_2_ photocatalysts with ultrasonic technology. Their experimental results showed that the degradation rate of phenolic groups in lignin was as high as 93% within 180 min. It is worth mentioning that the degradation rate also showed an upward trend with the increase in ultrasonic frequency. These research results strongly demonstrate the great potential and application prospects of sono-photocatalytic technology in depolymerizing and converting lignin into high-value chemicals.

**Figure 7:**
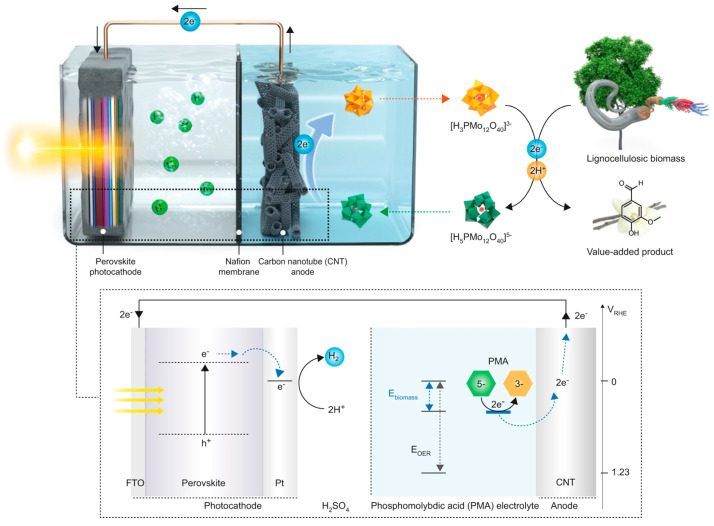
Schematic diagram of the principle of photoelectric synergistic catalytic biomass hydrogen production [87].

Similar to photocatalysis, electrocatalytic strategies have also attracted much attention for their high efficiency and environmental protectiveness. Combining photocatalysis with electrocatalysis can not only effectively suppress the recombination rate of photogenerated electron–hole pairs on the surface of photocatalysts, improving catalytic efficiency, but also promote the electrochemical oxidation process, thereby accelerating the depolymerization of lignin. Choi et al. [87] developed a biomass-assisted solar hydrogen production system (Figure 7). The authors used halide perovskites as photocathodes and wood cellulosic biomass (LC) as a low-potential electron source to produce hydrogen. At the same time, they also introduced phosphomolybdic acid (PMA) as a soluble catalyst to more effectively extract electrons from biomass. Under simulated solar illumination conditions, PMA showed the ability to selectively depolymerize lignin in wood cellulosic biomass, producing value-added chemicals such as vanillin and acetyl vanillone. What is even more gratifying is that in this process, the structure of the cellulose and hemicellulose remains intact, which provides the possibility for further utilization in the future.

Overall, the combination of the photocatalytic depolymerization strategy and other technical methods undoubtedly has great development potential. In future research, how to further optimize this combined strategy to improve the depolymerization effect of lignin will undoubtedly become a new and challenging research focus.

## 6. Electrocatalytic Lignin Depolymerization

Electrochemical technology is widely regarded as one of the advanced oxidation processes with significant industrial application potential [88]. This technology occupies an important position in the field of catalysis due to its high efficiency and relatively low cost. It is favored for its environmental friendliness and easy handling. What is more worth mentioning is that it usually does not require additional expensive additives, which further reduces the application costs [89]. Under electrochemical catalysis, the depolymerization process of lignin is realized. This process involves electron migration under electrochemical conditions, which generates intermediate substances with strong redox properties in the reaction system. These intermediates then bind to lignin molecules to trigger depolymerization. In addition, lignin can be oxidatively depolymerized by directly implanting external field electrodes, which further enriches the means of electrochemical catalytic lignin depolymerization. In the process of the electrocatalytic depolymerization of lignin, the types and yields of depolymerization products can be affected by a variety of regulatory means. Specifically, by adjusting external factors such as the type of catalyst, the size of the current density, and the choice of electrode materials, the directional production of depolymerization products can be achieved. This feature makes electrochemical catalysis extremely flexible and practical in the field of lignin depolymerization. Similar to photocatalysis, electrocatalysis is also an efficient and environmentally friendly lignin depolymerization strategy. In the field of the value-added utilization of biomass products, both technologies have shown great development potential. In the future, combining electrocatalysis with other depolymerization strategies to further improve the depolymerization effect of lignin will undoubtedly be an effective strategy worthy of in-depth research and exploration.

### 6.1. Product Distribution of Lignin Electrochemical Depolymerization

The electrochemical depolymerization of lignin is a rather complex reaction process, which involves many homogeneous and heterogeneous electrochemical reactions that may compete with each other. The complexity of this process mainly stems from the diversity of bond types and the complexity of connection methods within lignin. In the electrolyte solution, the breaking and recombination of chemical bonds in the microscopic electrochemical reaction process of lignin are difficult to fully and clearly understand. In addition, the organic aromatic rings in lignin and the various functional groups on its side chains may affect the final product after the electrochemical depolymerization of lignin [90].

The design of the electrochemical reaction system is particularly important when considering external factors. Changes in experimental conditions such as the type of catalyst, the setting of current density, and the selection of electrode materials will have a significant impact on the type and yield of electrochemical depolymerization products. In most cases, the products obtained after lignin depolymerization are a mixture of various organic substances. However, through the thoughtful design of the electrochemical catalytic system and the optimization of parameter conditions, the directional production of value-added aromatic organic products can be achieved. Taking the study of Chen et al. [91] as an example, they used Pb/PbO_2_ as a catalyst in an alkaline solution to oxidize and decompose wheat straw lignin (WSL), and carried out reduction reactions on cathode alloy materials with different catalytic activities. As a result, about 36–42 wt% of guaiacyl-type compounds, 25–33 wt% of syringyl-type compounds, and 11–18 wt% of phenol-type compounds were detected. Another study conducted by Du et al. [92] developed a novel proton exchange membrane (PEM) electrolysis process. They used polyoxymethylene (POM) or FeCl_3_ as the catalyst and charge transfer medium at the anode for depolymerizing kraft lignin (KL) into low-molecular-weight aromatic compounds such as phenol, guaiacol, 1,2-dimethoxybenzene, vanillin, benzoic acid, and phthalic anhydride at the anode, while generating hydrogen at the cathode. The study also found that compared with traditional alkaline water electrolysis, the electrolysis process using POM or FeCl_3_ could reduce energy consumption by 40%. These studies not only deepen our understanding of the electrochemical depolymerization process of lignin, but also provide a useful reference for the development of efficient and energy-saving lignin depolymerization technology.

### 6.2. Electrode Materials

In the electrocatalytic process, active intermediates are dynamically generated as the reaction proceeds and may actively participate in the electrochemical reaction. The concentration of these active intermediates can be effectively adjusted by precisely controlling the current density. The electrode not only plays the role of a catalyst in this process, but also is an important area for the reaction to occur and electrons to be received. Therefore, the progress of the reaction and the concentration of the product can be precisely controlled by adjusting the electrode material and current density, thereby improving the overall efficiency of the electrocatalytic reaction [93].

In the electrocatalytic system, electrodes are mainly divided into two categories: two- and three-dimensional electrodes. Among the two-dimensional electrodes, Pb-based electrodes have attracted much attention due to their high hydrogen evolution potential. Although Pb-based electrodes have a certain toxicity, their chemical stability, high conductivity, and high oxygen overpotential in corrosive electrolytes make them one of the popular electrodes in the study of lignin electrooxidation [94]. Compared with precious metals, the cost of Pb-based electrodes is relatively low, which further enhances their practical application value. Pan et al. [95] deposited PbO_2_ on TiO_2_ nanotubes by photochemical methods to prepare a stable Ti/TiO_2_NT/PbO_2_ electrode. Under the optimal conditions of 60 °C and 100 mA, the electrode can effectively depolymerize black liquor kraft lignin into vanillin and vanillic acid. Xu et al. [96] prepared FeCN–PbO_2_ electrodes by electrodeposition and applied them to the electrochemical degradation of alkali lignin (AL). The electrochemical characterization results showed that the FeCN–PbO_2_ electrode had a higher active surface area and oxygen evolution potential than the unmodified electrode, which was beneficial to improving its electrochemical oxidation ability. In addition, the service life of the FeCN-PbO_2_ electrode is significantly longer than that of the unmodified PbO_2_ electrode. Under the same conditions, the degradation rate of alkali lignin under the action of the FeCN-PbO_2_ electrode was as high as 52.25%, which was significantly higher than the 37.16% of the PbO_2_ electrode. Pb/PbO_2_ series electrodes have been widely used in the electrocatalytic depolymerization of lignin due to their excellent electrochemical properties. In fact, Pb-based electrodes are the mainstream electrodes in the current lignin electrochemical depolymerization technology.

In addition, Ni-based electrodes also occupy a place in the electrocatalytic depolymerization of lignin due to their excellent electrocatalytic performance and corrosion resistance. Under the action of Ni electrodes, water molecules in the solution first undergo an electron transfer process to form a highly reducing intermediate substance (Ni-Hads) on the metal surface, and then combine with organic matter adsorbed on the electrode to trigger the electrocatalytic reaction. Lan et al. [97] used Ni plates as cathodes and Pb/PbO_2_ electrodes as anodes to effectively depolymerize corn straw lignin in sodium hydroxide solution. The catalytic process can be divided into two key steps: electrocatalytic oxidation and hydrogenation. Oxidation products and smaller lignin molecular fragments are generated on the surface of the Pb/PbO_2_ anode, while the hydrogenation reaction of lignin fragments occurs specifically on the nickel cathode surface. The results showed that the electrode exhibited a good catalytic effect in lignin alkaline solution. Hads-like substances generated during the electrolysis process were adsorbed on the surface of the Ni electrode, which gives it a strong catalytic hydrogenation ability, allowing corn straw lignin to be converted into aromatic compounds through the electrocatalytic hydrogenation of the Ni cathode in the alkaline solution. Michael et al. [98] conducted in-depth research on the suitability of various commercially available transition metal alloys for the electrochemical catalytic depolymerization of lignin in alkaline media. They found that both Co- and Ni-based materials can selectively depolymerize lignin into vanillin with high yields. However, under the same electrochemical conditions, Ni foam electrodes exhibited a better corrosion resistance than Co metal electrodes. The Ni foam electrode activated by black liquor had a better electrochemical oxidation degradation performance for lignin in an alkaline solution, and its vanillin yield was 160% higher than that of ordinary Ni foam electrodes. Ni-based electrodes have become a reliable electrode material due to their excellent performance and efficient electrochemical properties, but their application is still not as extensive as Pb-based electrodes. This is mainly due to the relatively low content of metallic Ni in the earth, which leads to higher mining costs.

The three-dimensional electrode is an innovative design based on the traditional two-dimensional cathode and anode electrode structure. By fitting specific granular materials between the cathode and anode, a more complex electrode system is formed. Under the condition of applying voltage, these filled particles will be polarized into a large number of charged microelectrodes. A potential difference will be formed on the surface of each particle, with one end showing the characteristics of the anode and the other end showing the characteristics of the cathode. Therefore, this electrode structure is called a three-dimensional electrode. In the electrochemical process of three-dimensional electrodes, the depolymerization mechanism of macromolecules is particularly complex. It not only involves the basic principles of electrochemistry, but is also affected by multiple factors such as the initial depolymerization matrix, the selection of electrode materials, and the properties of granular electrode materials. These factors work together to affect the final depolymerization effect. Each particle fitted in the three-dimensional electrode can actually be regarded as a miniature electrolytic cell. In such a miniature electrolytic cell, direct electrochemical oxidation reactions can occur on the polarized particles, while indirect electrochemical oxidation may be achieved through strong oxidants generated in situ. This characteristic makes the three-dimensional electrode perform well in the catalysis and degradation of organic matter. It is worth mentioning that due to the introduction of these particle electrodes, the three-dimensional electrode system can usually surpass the traditional two-dimensional electrode system in electrochemical catalytic efficiency. However, as an emerging technology, the development history of three-dimensional electrodes is relatively short, and their stability in actual production applications still needs to be improved. Looking forward to the future, it is believed that with the deepening of research, the high efficiency and stability of three-dimensional electrodes will become the research focus in this field, laying a solid foundation for their application in a wider range of scenarios.

### 6.3. Combined Strategy for Electrocatalytic Lignin Depolymerization

In recent years, although significant progress has been made in the field of the electrocatalytic depolymerization of lignin, relying solely on electrocatalytic strategies for lignin depolymerization still faces the challenges of high energy consumption and unsatisfactory results [99]. To improve the efficiency of lignin depolymerization and point the way for future industrial applications, researchers have begun to explore new paths that combine electrocatalysis with other advanced technologies. For example, using ionic liquids as electrolyte solutions for lignin depolymerization has become an innovative method. Ionic liquids not only provide a unique solvent environment for chemical reactions, but also act as catalysts to effectively improve the selectivity and stability of reactions. Since lignin can be well dissolved in ionic liquids, this creates favorable conditions for the smooth progress of electrochemical reactions. In addition, ionic liquids also carry conductive functions, which help to efficiently transport protons in the reaction system, allowing lignin to undergo electrochemical reactions under relatively low voltage conditions. Reichert et al. [100] reported a new method in which they used a protonated ionic liquid, triethylammonium methanesulfonate, as a solvent, and selected ruthenium/vanadium/titanium mixed oxides as electrocatalysts for the electrocatalytic cracking of lignin. The results showed that in this electrocatalytic reaction, with ionic liquids as electrolytes, lignin can undergo oxidative depolymerization to produce important products such as benzaldehyde, m-methylbenzaldehyde, 3-furancarboxaldehyde, and vanillin.

In addition to ionic liquid technology, the enzyme electrodepolymerization strategy also shows great application potential. The bioenzymatic method has occupied a place in the field of lignin depolymerization with its high efficiency and environmental protectiveness. Manganese peroxidase, peroxidase, and laccase are three widely used lignin depolymerases in the combined use of electrochemical oxidation and bioenzymes, that is, first using electrochemical oxidation to destroy the stable bonding structure in lignin, clearing the obstacles for the subsequent bioenzymatic depolymerization, and then using bioenzymes to depolymerize lignin small molecules. At the same time, both photocatalytic and electrocatalytic depolymerization technologies have pollution-free and environmentally friendly properties. Integrating the two to achieve optoelectronic integrated lignin depolymerization technology is undoubtedly a promising option. The integrated application of multiple technologies indicates an important direction for the future development of lignin depolymerization technology.

Although electrochemical depolymerization technology has shown great application potential in the field of lignin depolymerization or modification, the limited service life of the electrode and the problem of electrode contamination during the polymerization process still restrict the widespread application of this technology. Similar to the photocatalytic depolymerization strategy, the electrocatalytic depolymerization strategy also faces challenges in industrialization. The high cost of electrode materials and catalysts is a problem that needs to be solved urgently. Given this, future research should focus on the development of electrode materials with a high activity, long life, and low economic cost. At the same time, it is also crucial to explore more efficient lignin depolymerization electrocatalysts.

## 7. Biological Depolymerization of Lignin

Among the many depolymerization technologies for lignin, many traditional methods often rely on harsh reaction conditions such as a high temperature and high pressure, and are accompanied by the production of environmentally harmful by-products. In sharp contrast, the biological method stands out for its mild and environmentally friendly characteristics. This method uses biological catalysts in nature, such as bacteria, fungi, and various enzymes, to depolymerize lignin. After long-term evolution and natural selection, these biocatalysts have formed a unique metabolic mechanism that can effectively cleave the chemical bonds inside lignin and further convert it into aromatic compounds. These aromatic compounds can not only be further converted into energy or high value-added products through a variety of pathways, but the entire conversion process is also environmentally friendly. For this reason, the biological method is regarded as a green and sustainable approach in the field of lignin depolymerization [101]. In addition, the introduction of biocatalysts has significantly improved the selectivity of the reaction, thereby effectively inhibiting the occurrence of adverse side reactions. Compared with other types of catalysts, the reaction conditions required by biocatalysts are milder, which not only reduces the high requirements for equipment during lignin depolymerization, but also significantly restrains the generation of by-products such as coke. For a long time, researchers have been committed to exploring and optimizing biocatalysts suitable for lignin depolymerization and biomass processing. These research results have been widely used and verified in many industrial fields such as food, papermaking, and detergents [102].

### 7.1. Fungi

As an important microorganism utilized in the field of lignin depolymerization and degradation research, fungi have the ability to secrete a variety of lignin depolymerases, which have efficient decomposition and degradation effects on lignin. According to the characteristics of their mechanism of degrading lignin, fungal systems can be further divided into three categories: soft rot fungi, white rot fungi, and brown rot fungi [103]. Among them, white rot fungi have a wide range of applications, such as increasing the content of cellulose by removing lignin components in biomass, increasing the production of biomethane, and effectively removing phenolic compounds from pollutants [104]. White rot fungi exhibit a strong lignin degradation ability, which is closely related to their ability to produce a series of extracellular oxidases, including lignin peroxidase, manganese peroxidase, and phenol oxidase. In the degradation process of lignin, these enzymes play a vital role through oxidation–reduction reactions. Specifically, lignin peroxidase is mainly responsible for oxidizing non-phenolic aromatic ring structures, manganese peroxidase focuses on cleaving the connection between alkyl and aromatic rings, and laccase can catalyze the cleavage reaction of aromatic rings inside lignin [105].

Although fungi show significant advantages in lignin depolymerization, from an industrial perspective, their depolymerization efficiency is still inferior to that of some chemical catalytic methods [106]. In addition, white rot fungi release oxidoreductases during the degradation process, which sometimes cause lignin fragments to repolymerize, thus becoming a problem that needs to be solved. In addition, like many microorganisms, fungi are also sensitive to changes in environmental conditions, which may limit their lignin depolymerization effects in specific environments. To overcome these limitations, future research can focus on the use of genetic engineering and proteomics technologies to optimize and screen fungal strains that can survive in extreme environments and efficiently perform lignin depolymerization tasks.

### 7.2. Bacteria

At present, although it is generally believed that bacteria are not as effective as fungi in depolymerizing lignin, and the types of bacteria that can effectively depolymerize lignin are relatively limited, bacteria show significant advantages in environmental adaptability. They can survive in harsh conditions and show a good adaptability to changing environmental conditions. In addition, bacteria also have a short growth cycle and high genetic stability, which makes bacteria more reliable for genetic manipulation. Based on these characteristics, researchers are actively exploring ways to enhance their lignin decomposition ability by optimizing bacteria. It is reported that Rhodococcus jostii RHA1 (R. Jostii RHA1 for short), as a highly viable bacterium, can survive in adverse environments such as nutrient deficiency and high cell density. More importantly, it can act on the β-O-4 bond of lignin with the assistance of dye-decolorizing peroxidase (DyPs) to produce vanillin [107]. In addition, the vanillin dehydrogenase gene is deleted from R. jostii RHA1 through gene editing technology, so that the mutant R. jostii RHA1 can accumulate vanillin in its cells. In addition to R. Jostii RHA1, Pseudomonas putida KT2440 is also considered to be an effective lignin-degrading bacterium. It is able to decompose lignin into small molecules and produce and accumulate polyhydroxyalkanoates (PHAs), providing a new source of raw materials for the production of bioplastics. By genetically modifying Pseudomonas putida KT2440, its physiological characteristics have been significantly improved, such as shortening the lag phase, increasing the biomass yield, and accelerating its growth rate.

Among known bacteria, some specific enzymes that can depolymerize lignin have been identified, such as dye-decolorizing peroxidase (DyP). This type of enzyme is involved in the oxidative depolymerization of lignin, aromatic dyes, and aromatic xenobiotics. In particular, DyP is a heme-containing peroxidase with a low redox potential and mainly acts on the dimers and trimers of lignin. In addition, bacteria can also secrete other types of oxidases, such as cytochrome P450, non-heme iron enzymes, and Mn/Cu-containing oxidases.

Although bacteria have shown significant potential in the depolymerization and conversion of lignin, their overall efficiency is still lower than that of fungi. Genetic modification provides new possibilities for the bacterial depolymerization and conversion of lignin. However, due to the lack of an in-depth understanding of the genetic information of bacteria, this technology cannot be applied to all species and has great uncertainty.

### 7.3. Enzymes

At present, the enzymes used for lignin degradation are all isolated and extracted from fungi or bacteria. These enzymes have shown an efficient conversion ability for lignin in in vitro experiments. The advantage of in vitro enzymatic reactions is that they can maximize the direct contact between the enzyme and the substrate, thereby reducing the reaction time [108]. In most cases, only a single enzyme species is used for in vitro enzymatic reactions of lignin model molecules. This is due to the complexity of the enzymatic system and the possibility of substrate competition when multiple enzymes coexist. Most enzymes are not specific for the depolymerization of lignin. According to the reaction environment and mechanism of action, lignin depolymerases can be divided into two major categories: peroxidases and laccases. Among peroxidases, lignin peroxidases (LiPs) and manganese peroxidases (MnPs) have been intensively studied. Recently, based on their multifunctionality, two new enzymes, multifunctional peroxidases (VPs) and dye decolorization peroxidases (DyPs), have been discovered.

Lignin peroxidase was originally discovered to be secreted by fungi and subsequently found in bacteria. This enzyme belongs to the class of heme-containing peroxidases, and its enzymatic reaction requires hydrogen peroxide to activate and catalyze non-phenolic and phenolic compounds. To complete its catalytic cycle, lignin peroxidase relies on resveratrol as an electron donor and cofactor. Due to its high redox potential, lignin peroxidase is regarded as a highly promising peroxidase. In addition to lignin depolymerization, this enzyme also plays a key role in biomass pretreatment and has attracted widespread attention for its excellent lignin removal effects. Manganese peroxidase (MnP) and lignin peroxidase (LiP) are similar in reaction mechanism, and both require oxidants to start the catalytic cycle. In the reaction, Mn^2+^ is oxidized to Mn^3+^, which, as a strong oxidant, can produce phenoxy radicals in lignin depolymerization, thereby accelerating the decomposition of lignin. Generally, manganese peroxidase mainly oxidizes phenolic compounds, but in the presence of additional Mn^2+^, it can also oxidize non-phenolic lignin model compounds. However, repolymerization was also observed when LiP and MnP were used to depolymerize lignin polymers.

Laccase, as an oxidase, is mainly derived from white rot fungi, but can also be found in some bacteria such as Bacillus subtilis [109]. It can use oxygen as an electron acceptor to oxidize phenolic compounds, converting these compounds into unstable phenoxy radical intermediates, which in turn trigger polymer cleavage. In addition to phenolic compounds, laccase can also work with mediators with an electron transfer ability such as 2,2’-azinobis (3-ethylbenzothiazoline-6-sulphonic acid) (ABTS) and 1-hydroxybenzotriazole (HBT) to oxidize non-phenolic compounds. With the help of mediators, laccase can depolymerize up to 90% of lignin. In addition, laccase also shows excellent lignin removal and conversion capabilities. Future research on laccase can start from the genetic level to improve its stability and reactivity.

Multifunctional peroxidase (VP) and dye decolorizing peroxidase (DyP) play a pivotal role in the depolymerization of lignin. Multifunctional peroxidase, mainly found in fungi, is called a bifunctional enzyme because of its similarity in molecular structure to lignin peroxidase (LiP) and manganese peroxidase (MnP) [110]. Unlike manganese peroxidase, multifunctional peroxidase can independently oxidize Mn^2+^. At the same time, it is similar to lignin peroxidase, which can oxidize substrates with higher redox potentials. In addition to playing a role in lignin depolymerization, the enzyme is also widely used in delignification, azo dye oxidation, and industrial wastewater treatment. Dye decolorization peroxidase is another type of peroxidase that has attracted much attention in the study of lignin depolymerization. Although its sequence and structure are different from other peroxidases, its catalytic performance and mechanism are quite similar, both of which use hydrogen peroxide and a medium to oxidize the substrate. Based on sequence characteristics, dye decolorization peroxidase can be divided into four categories. Types A, B, and C are widely present in bacteria, while Type D is mainly produced by fungi. In terms of activity, Types A and B are relatively low, while Types C and D show higher substrate oxidation activity. 

Bioenzyme use is an efficient lignin depolymerization strategy. Bioenzyme has high selectivity. The product obtained by using bioenzyme to depolymerize lignin has higher purity, which is conducive to the value-added utilization of the subsequent products. Bioenzyme causes little pollution to the environment and is a green depolymerization strategy. However, compared with traditional chemical depolymerization strategies, bioenzyme depolymerizes lignin slowly, resulting in high costs, and the enzyme may become inactivated during the reaction. Therefore, the technology of the bioenzyme depolymerization of lignin still presents certain difficulties in industrial applications and requires further research, development, and optimization.

Compared with other depolymerization strategies, the biological depolymerization strategy for lignin requires relatively mild conditions. Among them, the enzymatic method not only shows a higher reaction rate, but also has a higher specificity. Unfortunately, the cost of using biological enzymes is too high. At the same time, due to the complex structure of lignocellulose, a single enzymatic depolymerization strategy will produce a large amount of impurities, such as proteins and carbohydrates. The presence of these impurities increases the difficulty of product separation and purification. To overcome these challenges, it is necessary to continuously research new enzyme preparations, optimize the reaction conditions, improve separation and purification technologies, and explore new industrial application paths.

## 8. Conclusions

In today’s society, with the rapid development of the economy and the continuous increase in energy consumption, mankind is facing the severe challenge of the gradual depletion of fossil energy. Therefore, the exploitation and application of biomass energy have become an inevitable trend for sustainable development in the future. As a major research hotspot in the field of biomass energy, the depolymerization technology of lignin is of self-evident importance. Various advanced technologies have been used to achieve the efficient and pollution-free depolymerization of lignin for producing high value-added monomer chemicals and fuels, which have a far-reaching significance in promoting the optimization and upgrading of energy structures. When exploring more efficient depolymerization strategies, we should start from the perspective of industrial production and focus on whether the relevant depolymerization technology has the potential for industrialization.

In this review, the current lignin depolymerization strategies are mainly summarized in detail. Table 1 lists the comparison of the various methods. Although the existing depolymerization strategies have achieved certain results, there are still many challenges such as excessive energy consumption, difficulty in product separation, and environmental unfriendliness. In addition, due to its own limitations, a single depolymerization strategy can no longer meet the needs of industrial production at this stage. In order to effectively respond to these challenges, it is particularly important to apply different catalytic strategies in series to minimize the occurrence of the above problems. However, the combined application of different catalytic strategies increases the complexity of the system and places more stringent requirements on equipment and control conditions, which poses a challenge to future industrial production. Therefore, it is urgent to accelerate the development of low-cost, efficient, and pollution-free series depolymerization systems to promote their widespread application in industrial production. This is not only an urgent task facing the current energy field, but also an important way to achieve sustainable development.

## Figures and Tables

**Table 1:** A brief summary of various lignin depolymerization methods.

Methods		Advantage	Disadvantage	References
Thermochemical depolymerization	Pyrolysis Microwave-assisted lignin depolymerization	Simple and fast operation Efficient and easy to handle	Poor selectivity and many by-products High energy consumption	[30,31,43,45]
Chemical depolymerization			Corrosive equipment, environmental pollution	[47,49,51,55,56,62,64,67,68]
	Acid or base catalysts	Efficient and fast	High cost	
	Metal catalysts	High selectivity	High cost and difficult product separation	
	Ionic liquids	Highly adjustable reaction	Difficulty in recovery	
	Deep eutectic solvents	Strong designability	Difficulty in solvent recovery and product separation	
Photocatalytic depolymerization	Sole photocatalysis	Strong selectivity, low energy consumption, environmentally friendly	High catalyst cost and poor stability	[72,74,79,87]
Electrochemical depolymerization	Combined strategy for electrocatalytic lignin depolymerization	Strong selectivity, low energy consumption, environmentally friendly	High requirements for equipment, high cost, difficult industrialization	[88,89,92,95]
Biological depolymerization	Fungi Bacteria Enzymes	Environmentally friendly Environmentally friendly Environmental protection and directional depolymerization	Low efficiency Long cycle and high cost Long cycle, high cost, vulnerable to environmental impact	[101,102,106,107,108,109]
